# Local and global microarchitecture is associated with different features of bone biomechanics

**DOI:** 10.1016/j.bonr.2020.100716

**Published:** 2020-09-15

**Authors:** Jean-Paul Roux, Stéphanie Boutroy, Mary L. Bouxsein, Roland Chapurlat, Julien Wegrzyn

**Affiliations:** a INSERM UMR 1033, Université de Lyon, Lyon, France; b Orthopedic Biomechanics Laboratory, Beth Israel Deaconess Medical Center and Harvard Medical School, Boston, MA, USA; c Department of Orthopedic Surgery, Lausanne University Hospital – CHUV, Lausanne, Switzerland

**Keywords:** Osteoporosis, Fracture, Bone microarchitecture, Local structural weakness, Bone biomechanics, Micro-CT

## Abstract

**Purpose:**

Beside areal bone mineral density (aBMD), evaluation of fragility fracture risk mostly relies on global microarchitecture. However, microarchitecture is not a uniform network. Therefore, this study aimed to compare local structural weakness to global microarchitecture on whole vertebral bodies and to evaluate how local and global microarchitecture was associated with bone biomechanics.

**Methods:**

From 21 human L3 vertebrae, aBMD was measured using absorptiometry. Parameters of global microarchitecture were measured using HR-pQCT: trabecular bone volume fraction (Tb.BV/TV_global_), trabecular number, structure model index and connectivity density (Conn.D). Local minimal values of aBMD and Tb.BV/TV were identified in the total (Tt) or trabecular (Tb) area of each vertebral body. “Two dimensional (2D) local structural weakness” was defined as Tt.BMD_min_, Tt.BV/TV_min_ and Tb.BV/TV_min_. Mechanical testing was performed in 3 phases: 1/ initial compression until mild vertebral fracture, 2/ unloaded relaxation, and 3/ second compression until failure.

**Results:**

Initial and post-fracture mechanics were significantly correlated with bone mass, global and local microarchitecture. Tt.BMD_min_, Tt.BV/TV_min_, Tb.BV/TV_min_, and initial and post-fracture mechanics remained significantly correlated after adjustment for aBMD or Tb.BV/TV_global_ (*p* < 0.001 to 0.038). The combination of the most relevant parameter of bone mass, global and local microarchitecture associated with failure load and stiffness demonstrated that global microarchitecture explained initial and post-fracture stiffness, while local structural weakness explained initial and post-fracture failure load (*p* < 0.001).

**Conclusion:**

Local and global microarchitecture was associated with different features of vertebral bone biomechanics, with global microarchitecture controlling stiffness and 2D local structural weakness controlling strength. Therefore, determining both localized low density and impaired global microarchitecture could have major impact on vertebral fracture risk prediction.

## Highlights

• Global and local microarchitecture were associated with different features of bone biomechanics.
• Localized low density and/or impaired microarchitecture regions could have major impact on bone mechanical behavior.
• Global microarchitecture determined initial and post-fracture vertebral stiffness.
• Local microarchitecture determined initial and post-fracture vertebral failure load.

## 1 Introduction

Osteoporosis is characterized by an increased fracture risk and operationally defined using dual-energy X-ray absorptiometry (DXA) measurement of areal bone mineral density (aBMD). It has been demonstrated, however, that the biomechanical evaluation of osteoporotic fracture risk was improved by a global approach of exploring “bone strength”, that includes various averaged parameters of the whole volume of interest such as bone geometry, microarchitecture and matrix properties, rather than aBMD measurement alone (Sornay-Rendu et al., 2005; Siris et al., 2004; Bouxsein, 2005; Lester, 2005). Many clinical and ex-vivo studies have examined the contribution of trabecular and cortical microarchitecture to bone biomechanics (Roux et al., 2010; Wegrzyn et al., 2010; Wegrzyn et al., 2011; Boutroy et al., 2005; Sornay-Rendu et al., 2007; Burghardt et al., 2011; Banse et al., 2001; Hulme et al., 2007). Using an observational approach, Banse et al. reported a marked heterogeneity in the vertebral trabecular microarchitectural network with the anterior region of the vertebral body being more deteriorated compared to the posterior region (Banse et al., 2001). In addition, the vertebral body mechanical behavior was demonstrated to differ along with variations in trabecular microarchitecture within vertebral regions (Wegrzyn et al., 2010; McCubbrey et al., 1995). Commonly, the anterior part of the lumbar vertebral body appeared to be more strongly related to the vertebral failure load and, probably, the best region to explore when predicting the vertebral fracture risk (Hulme et al., 2007). Therefore, this heterogeneity of trabecular microarchitecture within the vertebral body suggested the importance of local effects of structural weakness on its mechanical behavior (Wegrzyn et al., 2010; Banse et al., 2001; Hulme et al., 2007). Only a few biomechanical or high-resolution finite element model studies have analyzed the mechanical effects of local variations in trabecular microarchitecture on whole bone specimens to bring new insights in the understanding of the interplay between local and global microarchitecture to mechanical behavior (Nazarian et al., 2006; Perilli et al., 2008; Costa et al., 2017; Jackman et al., 2016; Crawford et al., 2003; Goff et al., 2015). These studies emphasized that whole bone failure was controlled by local mechanical effects potentially attributable to local structural weakness (Nazarian et al., 2006; Perilli et al., 2008; Costa et al., 2017; Jackman et al., 2016; Crawford et al., 2003; Goff et al., 2015). However, most of these previous studies were performed on trabecular bone samples or biopsies with average measurements across the whole bone specimen even though trabecular microarchitecture is not uniformly distributed throughout a vertebra and have not investigated local structural weakness (Roux et al., 2010; Wegrzyn et al., 2010; Wegrzyn et al., 2011; Boutroy et al., 2005; Sornay-Rendu et al., 2007; Burghardt et al., 2011; Banse et al., 2001; Hulme et al., 2007).

Taken altogether, these results provide a strong rationale for exploring the contribution of local variations in trabecular microarchitecture on the whole bone mechanical behavior. Therefore, the aims of this study were to: 1/ evaluate local structural weakness in microarchitecture across the whole vertebral body, 2/ compare local structural weakness to global microarchitecture and 3/ assess how local and global bone mineral density and microarchitecture were associated with initial and post-fracture mechanical behavior. We hypothesized that local and global trabecular microarchitecture was associated with different features of bone biomechanics (i.e., elastic or plastic mechanical properties) and, thereby, could improve the understanding and prediction of vertebral fracture risk.

## 2 Material and methods

### 2.1 Bone specimens and bone density assessment

Twenty-one L3 vertebrae were harvested fresh from 21 whole lumbar spines (L1 to L5) of anonymized human donors (11 men, mean age = 75 ± 10 years and 10 women, mean age = 76 ± 10 years), that were already used for other types of experiments in previous studies (Roux et al., 2010; Wegrzyn et al., 2010; Wegrzyn et al., 2011). Source of the donors was anatomic donation dedicated to education and research. According to the French regulation, IRB approval was not required at the time of the study. However, written informed consent of the patients was obtained before death. L3 vertebra was chosen as a model regarding both its anatomical location at the top of the lumbar lordosis and with horizontal and parallel endplates in the anatomical standing position. Therefore, the uniaxial compressive mechanical testing performed in this study was likely to be consistent with the L3 mechanical loading in vivo. Specimens were obtained fresh and maintained frozen at −20 °C wrapped in a saline-soaked gauze until mechanical testing. The absence of prevalent fractures or significant bone diseases (i.e., bone metastasis, Paget's disease of bone, or major osteoarthritis) involving the whole lumbar spine was confirmed using high resolution lateral radiographs (Faxitron X-Ray Corporation, Lincolnshire, IL USA) prior to L3 dissection. Severity of lumbar osteoarthritis (OA) was assessed according to the Kellgren-Lawrence (K/L) grading scale. Vertebrae with severe OA (grade 4) were excluded. Among vertebrae included in the study, 11 (52%), 8 (38%), and 2 (10%) were graded normal, minimal, or moderate OA, respectively. Whole vertebral body aBMD* (g/cm^2^) and bone mineral content (BMC*, g) were measured using DXA (Delphi W®, Hologic, Waltham, MA USA) in the lateral projection through a 42-mm thick Plexiglas plate to mimic soft tissue attenuation.

### 2.2 Assessment of bone microarchitecture

**Fig. 1:**
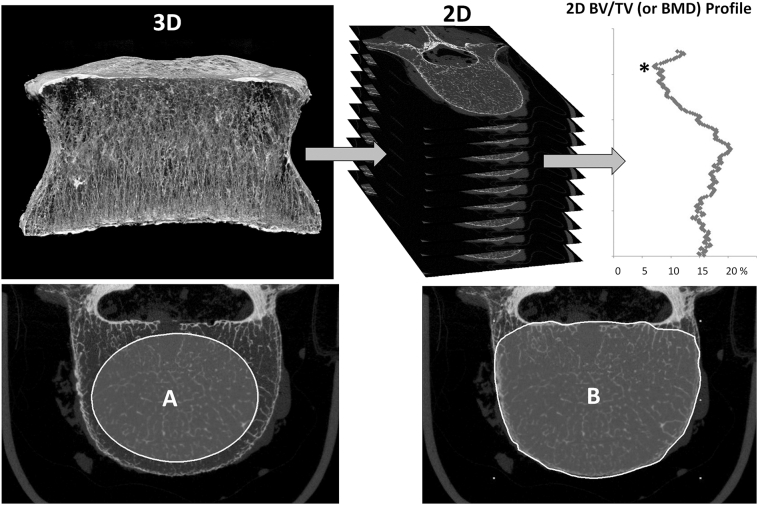
Measurement of the local structural weakness corresponding to the two-dimensional (2D) minimum value (*) in Tt.BV/TV_min_, Tb.BV/TV_min_ and Tt.BMD_min_ across the 2D stack of slices reconstructed along the normal cranio-caudal axis of the vertebral body. A: Delimitation of the trabecular region of interest (Tb), B: total region of interest (Tt) including cortical shell.

Three-dimensional (3D) bone microarchitecture was measured using a high-resolution peripheral quantitative computed tomography system (HR-pQCT; XtremeCT®, Scanco Medical AG, Brüttisellen, Switzerland) with a nominal isotropic voxel size of 82 μm using the standard in vivo protocol (1536 × 1536 pixels, X-ray source: 60 kV, 900 μA, plates of 0.3 mm Cu and 1 mm Al filter soft X-rays in order to minimize beam hardening on 750 projections at 100 ms integration time per rotation). Vertebral bodies were scanned in a plastic bag. A phantom containing 5 rods of hydroxyapatite (HA) (0, 100, 200, 400 and 800 mg HA/cm^3^) embedded in a soft-tissue equivalent resin (QRM, Moehrendorf, Germany) was used to convert attenuation values to equivalent HA densities. The global threshold used for segmentation of the trabecular bone (188 mg HA/cm^3^) tended to slightly overestimate the trabecular structure in order to maintain intact the structure connectivity at the low resolution of 82 μm. Regarding the trabecular volume of interest, three ellipsoidal regions of interest were defined on the superior, central and inferior slices at two to four millimeters distant from the cortices. Then, the trabecular bone volume of interest (Fig. 1A) was automatically interpolated from these three ROIs. Concerning the total region of interest, a custom processing script using the shrink-wrap function (CTan, Skyscan Bruker, Aartselaar, Belgium) limited the region of interest to the outside borders of the cortical bone. Then, posterior arches were interactively removed to obtain the total volume of interest (Fig. 1B). The following parameters of trabecular microarchitecture were measured: trabecular bone volume fraction* (Tb.BV/TV_global_, %), trabecular number* (Tb.N, 1/mm), structure model index* (SMI) and connectivity density (Conn.D, 1/mm^3^). From each vertebral body, the image stack was extracted from the HR-pQCT images and strictly reoriented along the cranio-caudal mechanical axis corresponding to the mechanical loading of the vertebral body using Dataviewer and CTan software (Skyscan Bruker, Aartselaar, Belgium). From two-dimensional (2D)-slices, the minimal values of BMD and BV/TV were measured in the total (Tt) or trabecular (Tb) areas across the image stack (Fig. 1). The following parameters associated to “2D local structural weakness” were then defined as Tt.BMD_min_, Tt.BV/TV_min_ and Tb.BV/TV_min_ (Fig. 1).

### 2.3 Mechanical testing

**Fig. 2:**
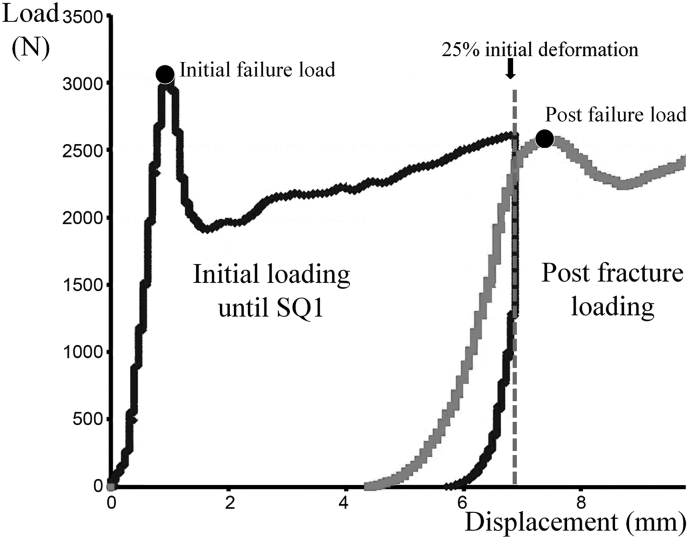
Load-displacement curves illustrating the 3 phases of the L3 vertebra mechanical testing. The black curve corresponded to the initial mechanical compression until SQ1 fracture (25%-deformation). The grey curve corresponded to the post-fracture mechanical compression performed after a 30 min-period of unloaded relaxation.

As reported in our previous studies, vertebral bodies were maintained moist at +4 °C in Ashman's solution (Roux et al., 2010; Wegrzyn et al., 2010; Wegrzyn et al., 2011). Before testing, soft tissues and posterior vertebral arches were removed. A polyester resin interface was applied to each endplate of the vertebral body using a device with two moving parallel trays to achieve strict parallel surfaces for load application. Then quasi-static uniaxial compressive testing was performed on the whole vertebral body submerged in Ashman's solution at controlled +37 °C. Mechanical preconditioning was performed prior to testing (10 cycles with loading at 100 N and unloading at 50 N). Then, mechanical testing was performed on the whole vertebral body in 3 phases using a screw-driven machine (Schenck RSA-250, Darmstadt, Germany) with a constant and controlled quasi-static uniaxial compressive displacement of 0.5 mm/min using a 5000-N load cell (TME, F 501 TC) and a displacement transducer mounted directly on the vertebral resin endplates (Roux et al., 2010; Wegrzyn et al., 2010; Wegrzyn et al., 2011). The initial phase compressed the vertebra to create a mild vertebral fracture (25%-deformation). This 25%-initial deformation corresponded to the grade I of semiquantitative (SQ1) assessment of vertebral fractures described by Genant et al. which is the most common grade of osteoporotic vertebral fracture at diagnosis (Genant et al., 1993; Kopperdahl et al., 2000). During the second phase, a 30-min unloaded period of relaxation was allowed to the vertebral body to recover from the initial deformation (Wegrzyn et al., 2011). During the third phase, the vertebra was compressed until failure to assess the vertebral mechanical behavior after sustaining an initial deformation (i.e., post-fracture mechanical behavior) (Fig. 2) (Wegrzyn et al., 2011).

The following initial and post-fracture mechanical parameters were measured: initial and post-fracture failure load* (N), and initial and post-fracture compressive stiffness* (N/mm) (Fig. 2).

Parameters above marked with * were already measured in prior analyses (Wegrzyn et al., 2010; Wegrzyn et al., 2011). They are presented again so that the statistical modeling can involve all relevant covariates.

### 2.4 Statistical analysis

Shapiro-Wilk tests were used to assess and confirm the normality of all the variable distributions. Data are presented as the mean, standard deviation, and range. The following tests were performed: 1/ unpaired *t*-tests to determine differences between male and female donors, 2/ Pearson correlation coefficients to determine relationships among variables, 3/ partial correlations with adjustment for global bone density parameters, and 4/ stepwise backward multiple regression models to define the most relevant bone mass, global microarchitectural and local structural weakness parameters explaining the mechanical testing outcome. Results were considered significant if *p* < 0.05. All statistical analyses were performed using IBM SPSS Statistics 22 software (IBM, Armonk, NY, USA).

## 3 Results

Descriptive statistics of absorptiometry, global microarchitecture, local structural weakness and mechanical parameters are presented in Table 1. No significant effect of sex was detected except for BMD, which was higher in males than in females (0.67 g/cm^2^ ± 0.12 versus 0.57 g/cm^2^ ± 0.10, *p* = 0.049). No significant difference was detected between sexes in terms of vertebral heights (29.80 mm for male vs 30.66 mm for female, *p* = 0.53) and Archimedes' vertebral volumes (55.73 cm^3^ for males vs 48.15 cm^3^ for females; *p* = 0.15). No significant effect of age was detected except for the initial failure load (*r* = −0.57, *p* = 0.008).

**Table 1:** Descriptive statistics of absorptiometry, global microarchitecture, 2D local structural weakness and mechanical parameters.

	mean **±** SD	Range
**Absorptiometry (DXA)**		
aBMD (g/cm^2^)	0.62 ± 0.12	(0.36–0.80)
BMC (g)	6.79 ± 1.91	(2.96–9.68)
**Vertebra morphometry**		
Vertebral height (mm)	30.2 ± 3.0	(26.4–37.5)
Archimedes volume (cm^3^)	52 ± 11	(34–80)
**Global microarchitecture**		
Tb.BV/TV_global_ (%)	13.49 ± 5.85	(3.99–23.54)
Conn.D (1/mm^3^)	0.61 ± 0.33	(0.11–1.28)
SMI (#)	2.61 ± 0.53	(1.47–3.33)
Tb.N (1/mm)	0.75 ± 0.16	(0.46–1.01)
**2D local structural weakness**		
Tt.BV/TV_min_ (%)	17.27 ± 6.60	(6.10–29.41)
Tb.BV/TV_min_ (%)	8.99 ± 4.80	(2.15–18.19)
Tt.BMD_min_ (g/cm^2^)	0.085 ± 0.031	(0.023–0.148)
**Mechanics**		
Initial failure load (N)	2615 ± 1136	(651–5481)
Initial stiffness (N/mm)	2938 ± 1585	(663–6741)
Post-fracture failure load (N)	2274 ± 892	(566–3916)
Post-fracture stiffness (N/mm)	1223 ± 556	(156–2310)

Descriptive statistics of absorptiometry, global microarchitecture, 2D local structural weakness and mechanical parameters (aBMD: areal bone mineral density, BMC: bone mineral content, Tb.BV/TV_global_: global trabecular bone volume fraction/tissue volume, Conn.D: connectivity density, SMI: structure model index, Tb.N: trabecular number, and Tt.BMD_min_, Tt.BV/TV_min_ and Tb.BV/TV_min_: minimal values of BMD and BV/TV measured in the total (Tt) or trabecular (Tb) areas across the whole vertebral body image stack).

**Fig. 3:**
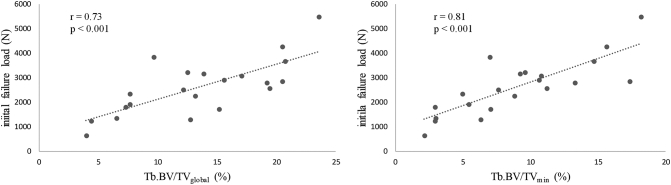
Scatter plots of global (Tb.BV/TV_global_) and local measurements (Tb.BV/TV_min_) vs initial failure load.

Bone density parameters (i.e., aBMD and Tb.BV/TV_global_) were significantly and positively correlated with initial and post-fracture failure load and stiffness (*r* = 0.54 to 0.86, *p* < 0.001 to 0.01) (Table 2). Global trabecular microarchitecture parameters (i.e., Conn D, SMI and Tb.N) were significantly correlated with initial and post-fracture mechanical behavior (*r* = 0.51 to 0.86, *p* < 0.001 to 0.02 for Conn d and Tb.N; *r* = −0.85 to −0.66, *p* ≤ 0.001 for SMI) (Table 2). Local structural weakness parameters (i.e., Tt.BMD_min_, Tt.BV/TV_min_ and Tb.BV/TV_min_) were significantly and positively correlated with initial and post-fracture mechanical parameters (*r* = 0.60 to 0.89; *p* < 0.001 to 0.004) (Table 2, Fig. 3). Tt.BMD_min_, Tt.BV/TV_min_ and Tb.BV/TV_min_ were also significantly and positively correlated with all the global trabecular microarchitecture parameters (*r* = 0.75 to 0.96, *p* < 0.001) (Table 3). Importantly, Tt.BMD_min_, Tt.BV/TV_min_ and Tb.BV/TV_min_ remained significantly and positively correlated with initial and post-fracture failure load after adjustments for aBMD (*r* = 0.56 to 0.77; *p* < 0.001 to 0.011) (Table 4).

**Table 2:** Pearson coefficients of correlation between bone mass, global microarchitecture, 2D local structural weakness, and initial and post-fracture mechanical parameters.

	Initial failure load	Initial stiffness	Post-fracture failure load	Post-fracture stiffness
**Bone mass and global microarchitecture**				
aBMD	0.66***	0.54*	0.72***	0.58**
BMC	0.54**	0.41	0.60**	0.44
Tb.BV/TV_global_	0.73***	0.65***	0.86***	0.84***
ConnD	0.70***	0.69***	0.80***	0.86***
SMI	−0.81***	−0.66***	−0.85***	−0.78***
TbN	0.51*	0.58***	0.67***	0.79***

**2D local structural weakness**				
Tt.BMD_min_	0.78***	0.66***	0.85***	0.77***
Tt.BV/TV_min_	0.81***	0.60**	0.89***	0.74***
Tb.BV/TV_min_	0.81***	0.66***	0.89***	0.79***

aBMD: areal bone mineral density, BMC: bone mineral content, Tb.BV/TV_global_: global trabecular bone volume fraction/tissue volume, Conn.D: connectivity density, SMI: structure model index, Tb.N: trabecular number, and Tt.BMD_min_, Tt.BV/TV_min_ and Tb.BV/TV_min_: minimal values of BMD and BV/TV measured in the total (Tt) or trabecular (Tb) areas across the whole vertebral body image stack.

* p<0.05; ** p<0.01; *** p<0.001

**Table 3:** Pearson coefficients of correlation between bone mass, global microarchitecture, and 2D local structural weakness parameters.

	aBMD	BMC	Tb.BV/TV_global_	ConnD	SMI	Tb.N	Tt.BMD_min_	Tt.BV/TV_min_
**BMC**	0.89***							
**Tb.BV/TV**_**global**_	0.79***	0.62**						
**ConnD**	0.73***	0.53*	0.94***					
**SMI**	−0.55**	−0.37	−0.87***	−0.78***				
**Tb.N**	0.72***	0.55**	0.86***	0.94***	−0.61**			
**Tt.BMD**_**min**_	0.89***	0.73***	0.93***	0.91***	−0.75***	0.85***		
**Tt.BV/TV**_**min**_	0.83***	0.67**	0.93***	0.86***	−0.82***	0.75***	0.96***	
**Tb.BV/TV**_**min**_	0.72***	0.55*	0.95***	0.89***	−0.91***	0.75***	0.91***	0.95***

aBMD: areal bone mineral density, BMC: bone mineral content, Tb.BV/TV_global_: global trabecular bone volume fraction/tissue volume, Conn.D: connectivity density, SMI: structure model index, Tb.N: trabecular number, and Tt.BMD_min_, Tt.BV/TV_min_ and Tb.BV/TV_min_: minimal values of BMD and BV/TV measured in the total (Tt) or trabecular (Tb) areas across the whole vertebral body image stack.

* p<0.05; ** p<0.01; *** p<0.001

**Table 4:** Partial correlations between 2D local structural parameters and initial and post-fracture mechanical behavior adjusted for areal bone mineral density (aBMD).

Controlling variable		Initial failure load (N)	Post-fracture failure load (N)
aBMD g/cm^2^	Tb.BV/TV_min_ (%)	r=0.64; p=0.002	r=0.77; p<0.0001
	Tt.BV/TV_min_ (%)	r=0.63; p=0.003	r=0.75; p<0.0001
	Tt.BMD_min_ g/cm^2^	r=0.56; p=0.011	r=0.65; p=0.003

Tb.BV/TV_min_, Tt.BV/TV_min_ and Tt.BMD_min_: minimal values of BMD and BV/TV measured in the total (Tt) or trabecular (Tb) areas across the whole vertebral body image stack

Using stepwise backward multiple regression models, the combination of the most relevant parameters of bone mass, global microarchitecture and local structural weakness was expressed by the equation “mechanical behavior = Tb.BV/TV_global_ + Conn D + Tb.BV/TV_min_ or Tt.BMD_min_ or Tt.BV/TV_min_”. This combination showed that global microarchitecture alone (i.e., Conn D) explained initial and post-fracture stiffness (*p* < 0.001) when global bone mass and local structural weakness parameters were removed from models in the second and third backward steps. In addition, local structural weakness alone (i.e. Tt.BMD_min_ or Tt.BV/TV_min_ or Tb.BV/TV_min_) explained initial and post-fracture failure load (*p* < 0.001) when global bone mass and global microarchitecture parameters were removed from models in the second and third backward steps (Table 5).

**Table 5:** Stepwise backward multiple regression models to define the most pertinent bone mass, global microarchitectural and local structural weakness parameters explaining the mechanical testing outcome.

	Model	p values		Model	p values	
*Dependent variable*	*Initial failure load*			*Post-fracture failure load*		
	**1**^**st**^**step**			**1**^**st**^**step**		
	Tb.BV/TV_global_	0.297	Out of model	Tb.BV/TV_global_	0.971	Out of model
	ConnD	0.591		ConnD	0.675	
	Tb.BV/TV_min_	0.015		Tb.BV/TV_min_	0.038	
	**2**^**nd**^**step**			**2**^**nd**^**step**		
	Tb.BV/TV_global_	0.339	Out of model	ConnD	0.558	Out of model
	Tb.BV/TV_min_	0.014		Tb.BV/TV_min_	0.002	
	**3**^**rd**^**step**			**3**^**rd**^**step**		
	Tb.BV/TV_min_	<0.0001		Tb.BV/TV_min_	<0.0001	
*Dependent variable*	*Initial stiffness*			*Post-fracture stiffness*		
	**1**^**st**^**step**			**1**^**st**^**step**		
	Tb.BV/TV_global_	0.459	Out of model	Tb.BV/TV_global_	0.687	Out of model
	ConnD	0.143		ConnD	0.123	
	Tb.BV/TV_min_	0.359		Tb.BV/TV_min_	0.854	
	**2**^**nd**^**step**			**2**^**nd**^**step**		
	ConnD	0.174	Out of model	Tb.BV/TV_global_	0.366	Out of model
	Tb.BV/TV_min_	0.576		ConnD	0.113	
	**3**^**rd**^**step**			**3**^**rd**^**step**		
	ConnD	<0.0001		ConnD	<0.0001	

Tb.BV/TV_global_: global trabecular bone volume fraction/tissue volume, Conn.D: connectivity density, Tb.BV/TV_min_: minimal values of BV/TV measured in trabecular areas across the whole vertebral body image stack.

## 4 Discussion

Even though the vertebral trabecular bone is not a uniform microarchitectural network, most of the previous studies evaluating the relationship between vertebral mechanical behavior and bone microarchitecture was based on averaged microarchitectural parameter measurements or microrachitectural heterogeneity assessment across whole bone specimens (Roux et al., 2010; Wegrzyn et al., 2010; Wegrzyn et al., 2011; Boutroy et al., 2005; Sornay-Rendu et al., 2007; Burghardt et al., 2011; Banse et al., 2001; Hulme et al., 2007; Hussein and Morgan, 2013; Hussein et al., 2013).

**Fig. 4:**
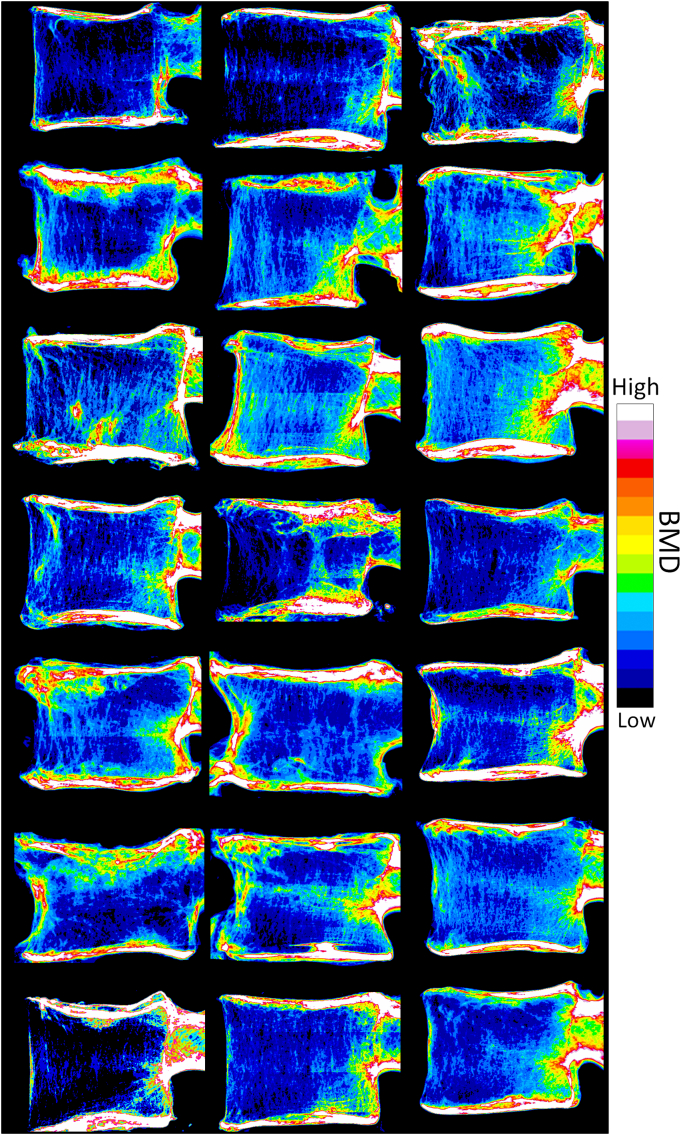
Density projection mapping of HR-pQCT bone mineral density illustrating areas of local structural weakness within the 21 vertebral bodies included in this study.

The current study was the first to evaluate the effects of global and local bone mineral density and microarchitecture on the initial and post-fracture mechanical behavior of a whole vertebral body (Fig. 4). The most important finding of this study was that global and local trabecular microarchitecture were associated with different features of bone mechanical behavior. We demonstrated that global microarchitecture was associated with initial and post-fracture stiffness (i.e., elastic properties) whereas local structural weakness was associated with initial and post-fracture strength (i.e., plastic properties). As previously reported by Liu et al., our results confirmed that global microarchitecture conditioned the elastic properties of vertebral trabecular bone (Liu et al., 2006). In addition, Jensen et al. demonstrated that, without changing the overall trabecular bone volume fraction, bone mechanical behavior dramatically varied from a uniform to irregular microarchitectural network (Jensen and Mosekilde, 1990). Therefore, local variations in bone microarchitecture leading to local structural weakness could be considered as a determinant factor for predicting localized failure as reported in the current study (Jensen and Mosekilde, 1990). Consequently, bone mass and global microarchitecture alone should not be considered as the unique indicators of trabecular bone mechanical competence (i.e., stiffness and strength).

The identification of the weakest link in microarchitecture and the evaluation of its contribution to the mechanical behavior of the whole bone specimen is therefore critical. Indeed, failure prediction could be improved using a “weakest link of the chain” approach. Local bone morphometry allowed identification of such “weakest” trabeculae and improved prediction of bone strength (Müller, 2003). For instance, Müller et al. demonstrated that a 10%-change in local trabecular thickness accounted for a 3-fold increase in mechanical strength (Müller, 2003). Previous ex-vivo studies also demonstrated that local variations in trabecular microarchitecture represented a determinant factor for localized regional failure and improved the mechanical behavior prediction of the whole bone specimen compared to global microarchitecture (Nazarian et al., 2006; Perilli et al., 2008; Costa et al., 2017; Jackman et al., 2016; Crawford et al., 2003; Goff et al., 2015). Using micro-CT, Nazarian et al. analyzed regional failure of vertebral biopsies among ten sub-regions and demonstrated that regional failure was related to local structural weakness areas (Nazarian et al., 2006). In the Nazarian's study, the weakest sub-region in term of minimal BV/TV corresponded to the sub-region where failure initially occurred (Nazarian et al., 2006). Therefore, determining the local structural weakness within the trabecular microarchitecture would improve the prediction of whole bone mechanical behavior (Nazarian et al., 2006). In addition, Perilli et al. measured local variations in trabecular microarchitecture (BV/TV_min_, Tb.Th_min_, Tb.Sp_min_, Tb.N_min_) on biopsies of human proximal femur to conclude that the evaluation of local minimal BV/TV (i.e. BV/TV_min_) improved failure prediction compared to the global BV/TV averaged on the whole bone specimen (Perilli et al., 2008). Importantly, the measurement of BV/TV_min_ was obtained and located within the region where failure occurred (Perilli et al., 2008). This emphasized that understanding the variations in mechanical behavior is improved when local minimal value of the microarchitectural parameters are considered, rather than averaged values on whole bone specimen. Nevertheless, these studies were performed on bone biopsies or samples that constituted a strong limitation, as the mechanical behavior of trabecular bone at any site and within site is dependent of the surrounding trabecular structure (Nazarian et al., 2006; Perilli et al., 2008). Indeed, with very low Tb.BV/TV values as observed in lumbar vertebrae from elderly donors, measuring 3D microarchitectural structure in isolated sub-regions remains questionable (Fyhrie and Schaffler, 1994; Hildebrand et al., 1999).

Compared to those studies, one of the major strengths of our study was to analyze the whole vertebral body to identify the weak cross-sections corresponding to the minimum value of bone density and trabecular microarchitectural parameters (Tt.BMD_min_ or Tt.BV/TV_min_ or Tb.BV/TV_min_) to define this so-called “local structural weakness”. The other strength was to evaluate the impact of global and local bone mineral density and microarchitecture on the vertebral mechanical behavior after a simulated vertebral fracture. Previously, Wegrzyn et al. (Wegrzyn et al., 2011) demonstrated that bone microarchitecture, but not bone mass, was associated with post-fracture mechanical behavior. Our current study strengthened these findings demonstrating the impact of “local structural weakness” not only on the initial but also on the post-fracture mechanical behavior. In addition, this measurement of local structural weakness was performed in re-orientated image stacks along a normal vertebral body cranio-caudal mechanical axis to simulate the main loading condition of L3 vertebrae in vivo. Indeed, Souzanchi et al. highlighted the importance to develop direction-dependent assessment of bone quality approach, as the trabecular network in whole bone specimen is oriented along with its mechanical solicitations in vivo (Souzanchi et al., 2012).

Our study had several limitations. The first limitation is the average age of our donors, therefore, our results might not be representative of the general population. However, the elderly population is the most susceptible to fragility fracture. Second, the loading mode used was quasi-static uniaxial compression. Since many osteoporotic vertebral fractures are anterior wedge fractures, more physiological testing conditions such as a rotating plate loading scenario may be of interest (Maquer et al., 2015). Other loading conditions that are relevant for vertebral fracture such as cyclic fatigue, bending or shearing may have different associations with bone mass or microarchitecture (Jackman et al., 2016). Third, partial volume effects, at a voxel sizes of 82 μm, could affect measurements, and a locally adaptive thresholding algorithm could be preferred to the use of classical global thresholding for microarchitecture measurements, which could increase the gap between low and high values. However, the minimal values of BV/TV were in agreement with the nonthresholding measurements of minimal values of BMD and most of clinical studies used global threshold. Fourth, the imaging resolution of 82-μm did not allow adequate evaluation of the thin cortical shell of the vertebral bodies. Therefore, the exact contribution of cortical shell alone to the initial and post-fracture vertebral mechanical behavior was not specifically assessed. However, the local structural weakness was evaluated not only on a trabecular bone volume of interest but also on the whole vertebral body that included the cortical shell.

In conclusion, this study was dedicated to the evaluation of the effect of global and local microarchitecture on the initial and post-fracture mechanical behavior on whole vertebral bodies and demonstrated that the global microarchitecture was associated with stiffness whereas local structural weakness was associated with strength. Therefore, determining trabecular bone regions of local structural weakness characterized by localized low density and/or impaired microarchitecture could have major structural impact on fracture risk prediction in clinical practice especially with the use of high-resolution quantitative computed tomography imaging devices.

## CRediT authorship contribution statement

**Jean-Paul Roux:** Methodology, Investigation, Formal analysis, Data curation, Writing - original draft, Writing - review & editing, Validation. **Stéphanie Boutroy:** Methodology, Investigation, Formal analysis, Data curation, Writing - original draft, Writing - review & editing, Validation. **Mary L. Bouxsein:** Methodology, Investigation, Formal analysis, Data curation, Writing - original draft, Writing - review & editing, Validation. **Roland Chapurlat:** Methodology, Investigation, Formal analysis, Data curation, Writing - original draft, Writing - review & editing, Validation. **Julien Wegrzyn:** Methodology, Investigation, Formal analysis, Data curation, Writing - original draft, Writing - review & editing, Validation.

## Declaration of competing interest

Jean-Paul Roux, Stephanie Boutroy, Mary L. Bouxsein, Roland Chapurlat and Julien Wegrzyn declare that they have no conflict of interest.
